# circNBPF10/miR-224 Axis Regulates PBX3 to Promote the Malignant Progression of Lung Cancer

**DOI:** 10.1155/2022/2832920

**Published:** 2022-03-17

**Authors:** Xuguang Zhao, Xiao Song, Yanxia Zhao

**Affiliations:** ^1^Clinical Laboratory, The People's Hospital of Shouguang, Weifang 262700, Shandong, China; ^2^Department of Clinical Laboratory, People's Hospital of Chiping District, Liaocheng, Shandong 252100, China; ^3^Department of Intensive Care Unit, People's Hospital of Changle County, Weifang, Shandong 262400, China

## Abstract

This study aims to reveal the potential effect of circNBPF10 on the malignant progression of lung cancer. The expression levels of circNBPF10 in lung cancer tissues and cell lines were detected via real-time quantitative PCR (RT-qPCR). The relationship between circNBPF10 expression and lung cancer metastasis was further analyzed. Effects on lung cancer cells after the knockout or overexpression of circNBPF10 were detected. Subsequently, the regulatory relationship of circNBPF10 with miR-224 was detected by using the dual-luciferase reporter gene. In addition, the role of pre-B-cell homeo box 3 (PBX3) in the progression of lung cancer affected by circNBPF10 was evaluated through a rescue experiment. circNBPF10 was highly expressed in lung cancer tissues and lung cancer cell lines. The expression level of circNBPF10 was significantly higher in patients with lung cancer and lymphatic metastasis or distant metastasis than in patients with nonmetastatic lung cancer. The downregulation of circNBPF10 reduced the proliferation, migration, and invasion of lung cancer cells. In lung cancer cells, circNBPF10 negatively regulated the expression of miR-224, whereas miR-224 directly targeted the expression of PBX3. The results of the rescue experiment confirmed that PBX3 was the key gene for the promoting effect of circNBPF10 on the malignant progression of lung cancer. circNBPF10 was highly expressed in lung cancer tissues and was associated with distant metastasis and poor prognosis in patients with lung cancer. circNBPF10 upregulated PBX3 by targeting miR-224 and promoted the malignant progression of lung cancer.

## 1. Introduction

The data reported in 2018 by the International Agency for Research on Cancer show that lung cancer is the most common cancer in the world (11.6% of total cases) and the leading cause of cancer-related deaths (18.4% of total cancer deaths) [1]. Non-small-cell lung cancer (NSCLC) accounts for 85% of the pathological types of lung cancer, while lung adenocarcinoma (LUAD) and lung squamous cell carcinoma are the most common subtypes. Small-cell lung cancer (SCLC), which is characterized by rapid doubling time and extensive early metastasis, has a 5-year survival rate of less than 7% due to the lack of methods for its early detection [2]. Most patients with SCLC only survive 1 year or less after diagnosis [3]. The active exploration of the pathogenesis of lung cancer indicates that the identification of new biomarkers related to this disease may be crucial for the treatment and prognosis of patients with this malignancy [4, 5].

CircRNAs are a class of noncoding RNAs. Memczak et al. identified 1950 human circRNAs, 1903 mouse circRNAs, and 724 nematode circRNAs through RNA sequencing. Numerous studies have confirmed that circRNAs play an important role in the occurrence and development of many human diseases, such as cancer [6], heart diseases [7], neurological diseases, diabetes, and immune system diseases [8]. In addition, an increasing number of studies have shown that circRNAs may be closely related to tumor proliferation, apoptosis, invasion, and metastasis. These pieces of evidence indicate the potential of circRNAs as new biomarkers and therapeutic targets and that the regulation of circRNAs may be an indispensable part of the mechanism, diagnosis, and treatment of lung cancer [9]. circRNAs can act as ceRNAs or microRNA (miRNA) sponges. ceRNAs that are composed of exons have a stable circular structure, and most contain miRNA response elements. Therefore, ceRNAs can act as efficient endogenous RNAs, effectively adsorbing miRNAs and preventing them from interacting with their target messenger RNAs (mRNAs) [10]. Bortoluzzi et al. reported that circNBPF10 is present in circulating peripheral blood in children with acute lymphoblastic leukemia (AML) [11]. circNBPF10 has been found to be upregulated in AML. However, the role of circNBPF10 in lung cancer has not yet been reported.

miRNAs are a class of noncoding RNA molecules with small relative molecular masses that are widely found in animals, plants, and viruses. The sizes of mature miRNA molecules range from 20 nt to 25 nt [12]. Currently, human cells contain ∼1000 miRNAs, among which ∼400 have been confirmed, that account for only ∼1% of protein-coding genes but regulate the expression of ∼30% of genes [13, 14]. Approximately 50% of miRNAs are located at fragile sites or regions that are amplified or absent in tumors [15]. miRNA expression profiles are closely related to the type, progression, survival rate, and prognosis of lung cancers [15]. miR-224 is a class of small noncoding RNAs that regulate the posttranscriptional translation of genes by binding seed sequences to the response elements of target mRNAs.

Pre-B-cell homeo box 3 (PBX3) is a homologous gene of the PBX family that contains TALE, which affects the transcription of downstream genes by interacting with the Hox protein to increase the DNA binding affinity of this protein [16]. Few studies have focused on the effect of PBX3, a homologous gene of PBX1, on the biological characteristics of tumors. PBX3 is highly expressed in gastric cancer, endometrial cancer, and other tumors. The expression of PBX3 in gastric cancer is closely related to the clinical stage, invasion depth, and differentiation degree of tumors, and the overexpression of PBX3 can promote the proliferation and clonal formation of gastric cancer cells [17]. miR-320d can affect EMT-related protein expression via the targeted inhibition of PBX3 [18].

The differential expression of circNBPF10 in lung cancer tissues was studied in this work. At the same time, the promoting effect of circNBPF10 overexpression on the malignant evolution of lung cancer and its molecular mechanism were studied. The molecular mechanism underlying the targeting of PBX3 by the circnBPF10/miR-224 axis was further verified via in vitro and in vivo experiments.

## 2. Methods

### 2.1. Patient and Lung Cancer Samples

A total of 15 patients who underwent lung cancer surgery from June 2017 to July 2019 at the People's Hospital of Changle County were selected. Tumor tissues and adjacent tissues (*n* = 15 each) were obtained during the operation. All patients did not receive radiotherapy or chemotherapy before operation, and their liver and kidney functions were normal. They had no infectious, immunity, endocrine, and circulatory system diseases. They also had no history of taking opioids and steroid hormones. The investigation was approved by the ethics committee of the People's Hospital of Changle County and was in line with the Declaration of Helsinki.

### 2.2. Cell Culture

Cell lines were purchased from the ATCC Cell Bank (Manassas, VA, USA). The human normal lung epithelial cell line BEAS-2B, the human SCLC line NCI-H446, the human large-cell lung cancer cell line NCI-H460, and the human NSCLC line A549 were cultured in 10% fetal bovine serum and RPMI-1640 medium. The culture fluid contained 100 U/mL of penicillin/streptomycin. The cells were cultured in an incubator containing 5% CO_2_ at 37°C.

### 2.3. Cell Transfection

si-circNBPF10, vector-circNBPF10, miR-224 mimics, miR-224 inhibitor, vector-PBX3, and corresponding control vectors were purchased from Guangzhou Gezi Biotech Co., Ltd. When the degree of cell adhesion reached 60%, the abovementioned plasmids were transfected into lung cancer cells in accordance with the instructions of Lipofectamine 2000 (Invitrogen, Carlsbad, CA, USA).

### 2.4. Dual-Luciferase Reporter Assay

The sequence of circNBPF10 or PBX3 mRNA containing miR-225 binding sites was amplified by PCR. Then, the sequence was inserted into the psiCHECK-2 luciferase reporter vector (Promega, Madison, WI, USA). Binding sites were mutated by using a quick-change site-directed mutagenesis kit (Agilent Technologies, Santa Clara, CA, USA). The luciferase reporter vector was transfected into cells that had been transfected with miR-225 mimics or NC mimics. After 48 h of transfection, luciferase activities were measured by using a dual-luciferase reporter assay system. All experiments were repeated three times.

### 2.5. Subcutaneous Xenograft Tumor Model

Female nude mice (18–20 g) aged 4–6 weeks old were purchased from Beijing Vital River Laboratory Animal Technology Co., Ltd. Lentivirus overexpressing circNBPF10 was transfected into A549 cells. The cells were injected subcutaneously into the backs of nude mice at a concentration of 1 × 10^6^. Each group comprised five nude mice. The injection volume was 100 *μ*L. The tumor size was measured every day and calculated by using the formula V = length × width^2^ × 0.5. After 1 month, the mice were euthanized with carbon dioxide, and the tumor tissues were removed and photographed. The tumor tissues were fixed for subsequent testing. The experiment was approved by the ethics committee of the People's Hospital of Changle County Hospital.

### 2.6. Immunohistochemistry

Paraffin sections were dewaxed for rehydration and repaired with an antigen repair solution. The sections were incubated with 3% H_2_O_2_ at room temperature for 5–10 min to eliminate endogenous peroxidase activity. The sections were rinsed with distilled water and soaked in phosphate buffered saline (PBS) for 5 min. The sections were blocked with 5% bovine serum albumin, treated with primary antibody (Ki-67, Abcam, 1 : 1000), and incubated at 4°C overnight. After being flushed with PBS, the sections were incubated with secondary antibodies at room temperature for 1 h. Slides were blocked with neutral balsam after DAB staining. The slides were observed and photographed under a microscope (Nikon, Japan).

### 2.7. TUNEL Staining

Paraffin-embedded tissue sections were dewaxed for rehydration and rinsed with PBS three times for 5 min each time. A protease K working solution was added to the sections, which were then reacted at 37°C for 30 min. Tissues were fixed at room temperature for 30 min, rinsed with PBS three times for 5 min each, immersed in the sealing solution, and sealed at room temperature for 10 min. The sections were rinsed with PBS three times for 5 min each time. Each sample was incubated with 100 *μ*L of TdT enzyme reaction solution at 37°C for 60 min in the dark. After DAPI staining, the slides were observed under a microscope (Nikon, Japan).

### 2.8. Real-Time Quantitative PCR Analysis

Total RNA was isolated by using TRIzol Reagent (Invitrogen). The cytoplasmic and nuclear RNAs of the cells were separated and extracted by using a Paris Kit (Invitrogen). First-strand cDNA was then generated by using a PrimerScript™ reagent kit (TaKaRa, Dalian, China). PCR amplification was carried out by using a SYBR Green PCR kit (TaKaRa) on an ABI PRISM 7300 Sequence Detection system (Applied Biosystems, Foster City, CA, USA). The PCR primer sequences were as follows: miR-224 primer: 5′-GAGCCCAAGTCACTAGTGGT-3′, downstream primer: 5′-GTGCAGGGTCCGAGGT-3′; U6: sense strand 5′-GGCTGGTAAGGATGAAGG-3′, antisense strand 5′-TGGAAGGAGGTCATACGG-3′; GADPH upstream primer: 5′-AGCCACATCGCTCAGACA-3′, downstream primer: 5′-TGGACTCCACGACGTACT-3′. Gene expression in each group was calculated in accordance with the 2^−ΔΔCT^ method. GAPDH was used as an internal reference to calculate relative total RNA expression, and U6 was used as an internal reference to calculate relative miRNA expression.

### 2.9. Western Blot Analysis

The SDS-PAGE gel was prepared and solidified at room temperature for 30 min. The electrophoresis tank was installed. The protein solution had a weight of 20–30 *μ*g. The gel was run under constant pressure. Electrophoresis was completed after 2 h. The film was transferred in constant current mode and subjected to wet rotation at 300 mA at 4°C for ∼0.5 h. The PVDF membrane was removed and immersed in a blocking solution for 1 h. The target protein band was cut in accordance with the label and incubated with diluted primary antibodies, namely, PBX3 (ab109173, 1 : 1000 dilution; Abcam, Cambridge, MA, USA) and GAPDH (Abcam), at 4°C overnight. The PVDF membrane was cleaned with TBST three times. The membrane was incubated with diluted secondary antibodies at room temperature for 45 min and then cleaned with TBST three times under shaking. The PVDF membrane was removed and placed in a gel imaging system. An image of the gel was developed, fixed, and saved.

### 2.10. Fluorescence In Situ Hybridization Assay

Lung cancer cells were immobilized, cleaned, and then incubated with prehybridization solution at 37°C for 1 h. A hybridization solution containing circNBPF10-Probe (8 ng/*μ*L) was dripped onto the cells. The cells were hybridized at 37°C and washed overnight. The sections were stained with DAPI dye, incubated in the dark for 8 min, rinsed, and blocked with an antifluorescent quenching agent. The processed sections were observed under an ECLIPSE Ci positive fluorescence microscope, and images were collected.

### 2.11. CCK-8 Assay

The cells were formulated into a cell suspension with a concentration of 3 × 10^4^ cells/mL and seeded into a 96-well cell culture plate. A total of 180 *μ*L of cell suspension was added to each well. Different plasmids were transfected into A549 and NCI-H446 cells at 24 h after cell culture. A total of 20 *μ*L of CCK-8 reagent was added to each well at 48 h after transfection. After incubation at 37°C for 4 h, an automatic microplate reader was used to read the absorbance at 450 nm. The cell survival rate was calculated as follows: (the value of the cell of each concentration in the experimental group/the value of the cell the blank control group) × 100%.

### 2.12. Transwell Invasion Assay

At 48 h after transfection, digested cells were centrifuged and suspended in a culture medium containing 0.1% serum. Cell density was adjusted to 1 × 10^5^/mL. A total of 500 *μ*L of the cell culture solution containing 10% FBS was added to the lower layer of a Transwell chamber, and 250 *μ*L of the cell suspension was added to the upper layer of the chamber. After 24 h of incubation at room temperature, the cells were removed from the upper layer of the chamber and stained with 0.1% crystal violet for 15 min. Five uniformly distributed fields of view under 200× magnification (Nikon, Japan) were selected to determine the number of transmembrane cells.

### 2.13. miRNA Pull-Down Experiment

Synthetic biotin-labeled miR-224 (bio-miR-224) was transfected into the cells by using Lipofectamine 2000. The cells were lysed on ice. Cell lysates were incubated with streptavidin agarose beads and then placed in an ice bath for 2 h. The cell lysis buffer was centrifuged. The circNBPF10 levels of bio-miR-224 pull-down were detected via QRT-PCR.

### 2.14. Scratch Test

The cells were seeded in 24-well plates at a concentration of 5.0 × 10^5^ cells/well. When the cell density reached 80%, an artificial wound was created in the fused cell monolayer by using a 100 *μ*L pipette tip. An inverted microscope was used to take images of wound closure at 0 (cell adhesion) and 48 h. Finally, the percentage of wound closure was calculated.

### 2.15. Statistical Analysis

SPSS 22.0 statistical analysis software was used. All data were repeated at least three times and expressed as the mean ± standard deviation. The *t*-test was performed to compare two groups, and a one-way ANOVA was performed to compare multiple groups. *P* < 0.05 was considered statistically significant.

## 3. Results

### 3.1. circNBPF10 Is Highly Expressed in Lung Cancer Tissues and Cell Lines

**Figure 1:**
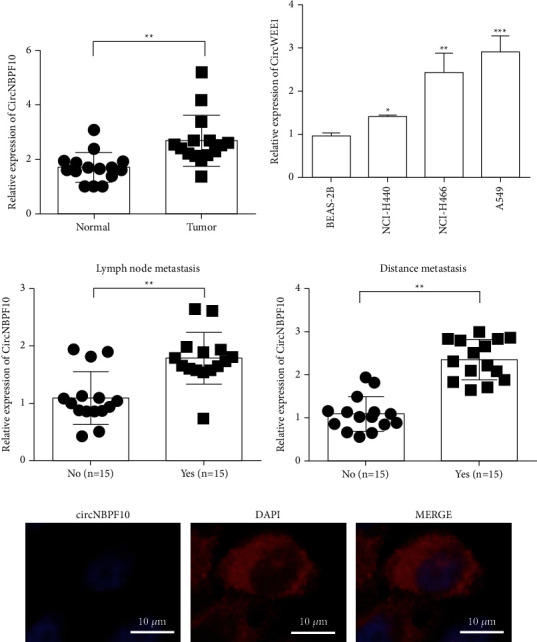
Expression of circNBPF10 in lung cancer and adjacent tissues and analysis of the clinical information of patients. (a) circNBPF10 levels in adjacent tissues and lung cancer tissues were detected via qRT-PCR. A total of 15 lung cancer tissues and 15 control tissues were analyzed. (b) circNBPF10 expression levels in normal lung epithelial cells and lung cancer cells were detected through qRT-PCR. (c) circNBPF10 levels in patients with lung cancer and with or without lymph node metastasis were determined via qRT-PCR. (d) circNBPF10 levels in patients with lung cancer and with or without distant metastasis were detected via qRT-PCR. (e) Fluorescence in situ hybridization was used to detect circNBPF10 expression and cell localization. ^*∗*^*P* < 0.05, ^*∗∗*^*P* < 0.01, and ^*∗∗∗*^*P* < 0.001.

First, we used real-time quantitative PCR (RT-qPCR) to detect the expression of circNBPF10 and found that circNBPF10 expression in lung cancer tissues was significantly upregulated relative to that in paracancer tissues (Figure 1(a)). Similarly, the expression level of circNBPF10 in lung cancer cells was significantly higher than that in normal lung cells (Figure 1(b)). Among the three lung cancer cell lines, A549 and NCI-H446 had the highest circNBPF10 expression levels. Therefore, A549 and NCI-H446 were used in subsequent experiments. circNBPF10 expression was associated with metastasis in patients with lung cancer. We found that the expression level of circNBPF10 in the lymphatic and distant metastasis groups had markedly increased relative to that in the nonlymphatic and distant metastasis groups (Figures 1(c) and 1(d)). The expression and subcellular localization of circNBPF10 were detected via fluorescence in situ hybridization. The results of this analysis showed that circNBPF10 was mainly expressed in the cytoplasm (Figure 1(e)).

### 3.2. Overexpression of circNBPF10 Promotes Tumor Proliferation

**Figure 2:**
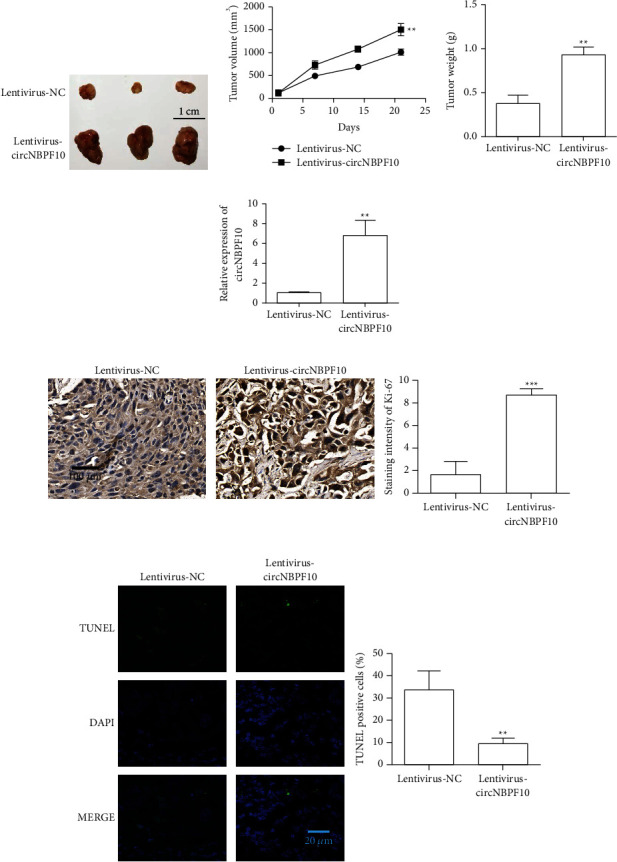
Overexpression of circNBPF10 promotes tumor progression. (a) Tumor volume detected after circNBPF10 overexpression (*N* = 5). (b) Tumor growth curve detected after the injection of A549 cells overexpressing circNBPF10. (c) Tumor weight detected after the overexpression of circNBPF10. circNBPF10 overexpression promoted tumor weight. (d) Detection of circNBPF10 expression in each group. (e) Ki-67 immunohistochemical staining results for each group. (f) TUNEL staining of tumors in each group. ^*∗∗*^*P* < 0.01 and ^*∗∗∗*^*P* < 0.001.

We further established a xenograft tumor model to study the effects of circNBPF10 on the proliferation of lung cancer. We first constructed the lentivirus overexpression vector of circNBPF10 (Figure S1) and then constructed the A549 cell line with stable circNBPF10 expression. Changes in tumor volume and weight were detected after the establishment of subcutaneous tumor-bearing cell lines with stable circNBPF10 expression. Experimental results showed that circNBPF10 overexpression could significantly increase tumor proliferation (Figure 2(a)and 2(b)) and tumor weight (Figure 2(c)). RT-qPCR analysis showed that transfection with the lentivirus overexpression vector could effectively upregulate the expression level of circNBPF10 in tumor tissues (Figure 2(d)). Subsequently, Ki-67 staining was used to detect the proliferation capability of the tumors. Experimental results showed that circNBPF10 overexpression increased tumor proliferation capability (Figure 2(e)). The TUNEL staining assay was used to detect the apoptosis of tumor tissues. The results showed that circNBPF10 overexpression inhibited tumor apoptosis (Figure 2(f)). We further investigated the effect of circNBPF10 on the clone formation of lung cancer cells. The results showed that the overexpression of circNBPF10 could promote the clonogenesis of lung cancer cells (Figure S2).

### 3.3. miR-224 Is the Target Gene of circNBPF10

**Figure 3:**
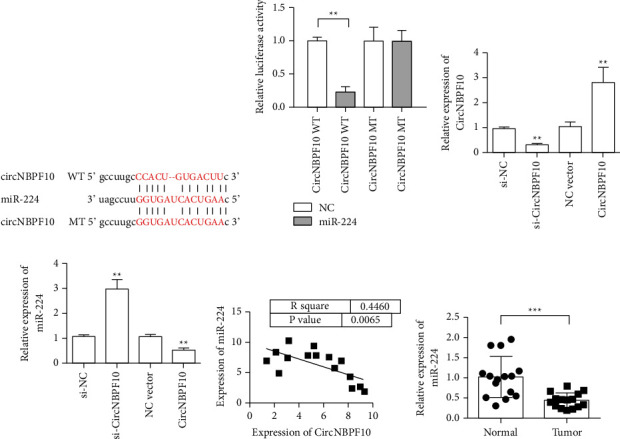
miR-224 is the target of circNBPF10. (a) Information map of binding sites between circNBPF10 and miR-224. (b) Dual luciferase detection of the binding of circNBPF10 to miR-224 (WT: wild type and MT: mutated type). (c) miRNA pull-down assay confirmed the regulatory relationship between circNBPF10 and miR-224. (d) circNBPF10 overexpression inhibited miR-224 expression. (e) Correlation analysis of circNBPF10 and miR-224 coexpression in lung cancer. (f) miR-224 expression in tumors was detected through qRT-PCR. ^*∗∗*^*P* < 0.01 and ^*∗∗∗*^*P* < 0.001.

We first analyzed the binding of circNBPF10 to miRNA to further investigate the mechanism of circNBPF10 in lung cancer. The analysis results showed that circNBPF10 could bind to miR-224 (Figure 3(a)). The binding of circNBPF10 to miR-224 was further confirmed by using the dual-luciferase reporter assay. The double luciferase reporter gene assay results showed that luciferase activity after the cotransfection of miR-224 mimics with pGL3-cirNBP10-WT plasmid had significantly decreased compared with that after the control treatment. The luciferase activity of the pGL3-cirNBP10-MT plasmid did not significantly change (Figure 3(b)). miRNA pull-down experiments further confirmed the regulatory relationship between circNBPF10 and miR-224. The experimental results showed that biotin-miR-224 could be enriched in circNBP10 effectively (Figure 3(c)). We overexpressed and downregulated circNBPF10 to verify the regulatory effect of circNBPF10 on miR-224. The expression of miR-224 was inhibited by the overexpression of circNBPF10 but was promoted by the reduction in circNBPF10 (Figure 3(d)). The coexpression of circNBPF10 and miR-224 was negatively correlated (Figure 3(e)). We further detected the changes in miR-224 expression in tumors. The experimental results showed that miR-224 in tumor tissues was downregulated relative to that in tissues from the control group (Figure 3(f)).

### 3.4. circNBPF10 Promotes the Malignant Evolution of Lung Cancer Cells

**Figure 4:**
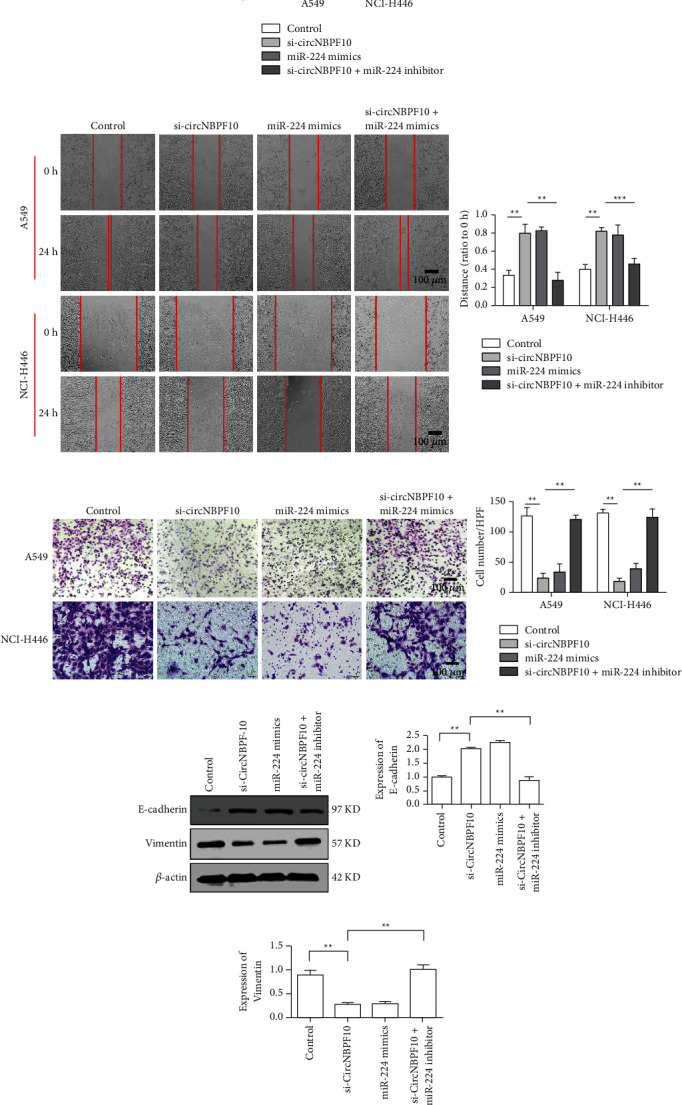
circNBPF10 adsorbs miR-224 through sponge action and promotes the malignant evolution of lung cancer cells. (a) Detection of cell proliferation in each group. (b) Detection of cell migration in each group. (c) Detection of cell invasion capability in each group. (d) Detection of E-cadherin expression in each group. (e) Detection of vimentin expression in each group (magnification ×200). ^*∗∗*^*P* < 0.01 and ^*∗∗∗*^*P* < 0.001.

The results of cell viability detection by CCK-8 showed that si-circNBPF10 inhibited the viability of A549 and NCI-H446 cells. Similarly, miR-224 mimics also inhibited cell proliferation. However, the miR-224 inhibitor was able to reverse the inhibitory effect of si-circNBPF10 on proliferation (Figure 4(a)). Transwell assay and scratch detection results showed that si-circNBPF10 and miR-224 mimics could inhibit the invasion and migration of lung cancer cells, whereas the miR-224 inhibitor could reverse the inhibition of si-circNBPF10 (Figures 4(b) and 4(c)). Results indicated that E-cadherin was upregulated in the si-circNBPF10 and miR-224 mimic groups, whereas vimentin expression was downregulated in the si-circNBPF10 and miR-224 mimic groups. However, the miR-224 inhibitor decreased the level of E-cadherin and increased that of vimentin relative to those in the si-circNBPF-10 group, suggesting that the downregulation of miR-224 could inhibit the EMT induced by circNBPF-1 (Figures 4(d) and 4(e)). All the abovementioned results showed that circNBPF10 promoted the malignant progression of lung cancer cells by sponging miR-224.

### 3.5. miR-224 Inhibits the Expression of PBX3

**Figure 5:**
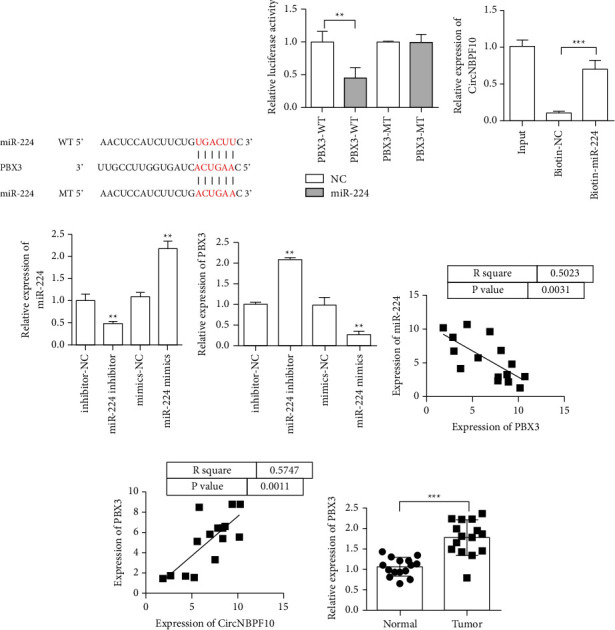
miR-224 inhibits PBX3 expression. (a) Information maps of miR-224 and PBX3 binding sites. (b) Dual luciferase reporter gene assay verified the combination of miR-224 and PBX3. (c) miRNA pull-down assay. (d) Verification of miR-224 expression. (e) miR-224 overexpression mimics inhibited the expression of PBX3. The content of PBX3 increased with the addition of the miR-224 inhibitor. (f) Correlation analysis of PBX3 and miR-224 coexpression. (g) Correlation analysis of circNBPF10 and PBX3 coexpression. (h) PBX3 expression in tumors detected by qRT-PCR. ^*∗∗*^*P* < 0.01.

We used TargetScan to predict the information of the target genes bound by miR-224 to study the target genes of miR-224. The results showed that miR-224 could bind to PBX3.  Figure 5(a) shows the map of the binding sites of miR-224 to PBX3. The binding of PBX3 to miR-224 was further confirmed via the double luciferase reporter assay (Figure 5(b)). We further confirmed the regulatory relationship between circNBP10 and miR-224 through miRNA pulldown. The experimental results showed that biotin-miR-224 could be enriched into circNBP10 effectively (Figure 5(c)). We overexpressed and downregulated miR-224 to verify the regulatory effect of miR-224 on PBX3 (Figure 5(d)). The detection results for PBX3 expression showed that the overexpression of miR-224 could inhibit the expression of PBX3, whereas the knockdown of miR-224 could promote the expression of PBX3 (Figure 5(e)). The coexpression of PBX3 and miR-224 showed a negative correlation (Figure 5(f)). The coexpression of PBX3 and circNBPF10 showed a positive correlation (Figure 5(g)). We further detected changes in PBX3 expression in tumors. The experimental results showed that PBX3 in tumor tissues was upregulated relative to that in control tissues (Figure 5(h)).

### 3.6. miR-224 Inhibits the Malignant Progression of Lung Cancer by Inhibiting the Expression of PBX3

**Figure 6:**
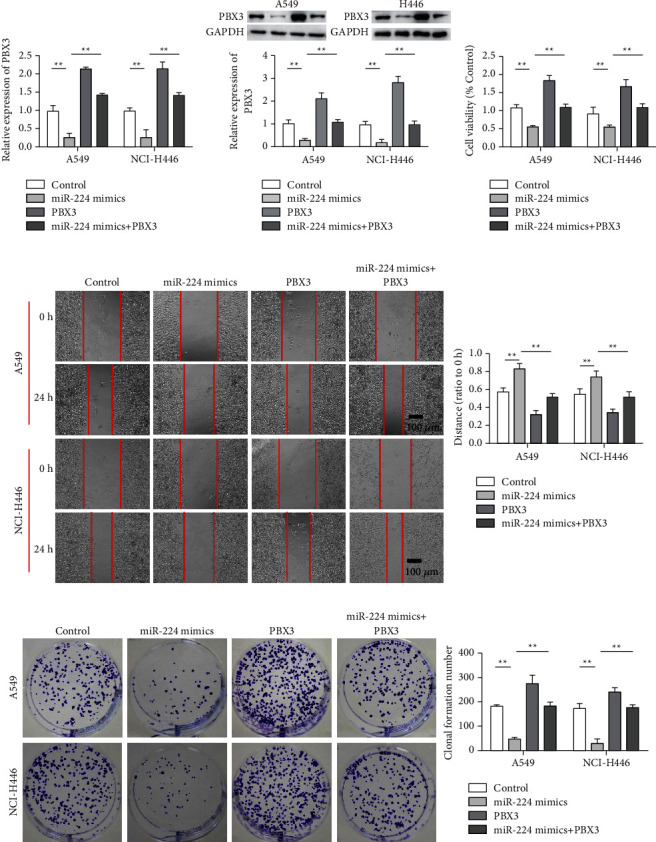
miR-224 reduces the malignant progression of lung cancer by inhibiting PBX3 expression. (a) Detection of PBX3 expression in each group via qRT-PCR. (b) Detection of PBX3 expression in each group via western blot analysis. (c) Detection of cell proliferation after different treatments. (d) Detection of cell migration after different treatments. (e) Cell clone formation results in each group (magnification ×200). ^*∗*^*P* < 0.05 and ^*∗∗*^*P* < 0.01.

RT-qPCR and western blot results showed that miR-224 inhibited PBX3 expression, whereas the overexpression plasmid increased the expression of PBX3, indicating that transfection was effective (Figure 6(a) and 6(b)). The CCK-8 assay showed that the viability of lung cancer cell lines decreased when miR-224 mimics were added. PBX3 overexpression promoted the viability of A549 and NCI-H466 cells. Meanwhile, PBX3 overexpression reversed the effect of miR-224 (Figure 6(c)). The results of cell migration and cell clone formation assays showed that the migration and clone formation capabilities of A549 and NCI-H446 cancer cell lines decreased upon treatment with miR-224 mimics. PBX3 overexpression promoted the migration and clone formation ability of A549 and NCI-H466 cells. Meanwhile, PBX3 overexpression reversed the effect of miR-224 on the migration and clone formation capabilities of lung cancer cells (Figures 6(d)–6(f)).

## 4. Discussion

Lung cancer is one of the cancers with the highest morbidity and mortality worldwide; it includes NSCLC, which accounts for ∼85% of all lung cancer cases [19–21]. Although some progress has been made in the treatment of NSCLC, the 5-year survival rate for lung cancer remains less than 15% [22–24]. Therefore, the study of molecular mechanisms and the discovery of new therapeutic targets are crucial to the treatment of lung cancer. This study aimed to describe the regulatory role of the circNBPF10/miR-224/PBX3 axis in lung cancer and to describe the cellular/molecular mechanisms of this role.

circRNA regulates the proliferation and progression of lung cancer by acting as a miRNA sponge. CRCRNF13 is downregulated by nearly 2.98 times in LUAD tumor tissue, and its expression level is significantly negatively correlated with tumor TNM stage and lymph node metastasis. Subsequent cytological analysis showed that CRCRNF13, which is located in the cytoplasm, can interact with the RNA-binding protein Ago2 by sponging miR-93-5p. Similarly, hsa_circ_00007385 is significantly upregulated in NSCLC tissues and lung cancer cells. In vitro experiments confirmed that it can significantly inhibit the proliferation, migration, and invasion of NSCLC cells by adsorbing miR-181 and significantly reduce the growth of gene knockout xenograft tumors. In addition, the expression of hsa_circ_0012263 in the circular RNA microarray of LUAD tissue is upregulated, and its expression level is related to tumor size. Cyclic RNA targets miR-22 via sponging the target Erb-B2 receptor tyrosine kinase 3 to promote the proliferation of LUAD cells. Furthermore, cirkMAN2B2 promotes the expression of FOXK1 by sponging miR-1275, thereby exerting a carcinogenic effect in lung cancer [25, 26]. The circ-BANP-mediated miR-503/LARP1 signaling pathway promotes the proliferation and metastasis of lung cancer [27]. Our experiment revealed several new discoveries: circNBPF10 was negatively correlated with miR-224 expression, with the former being upregulated and the latter being downregulated in lung cancer, and circNBPF10 overexpression significantly promoted the progression of lung cancer.

miRNAs can be used as tumor suppressors and cancer-promoting factors to regulate cell proliferation, apoptosis, invasion, metastasis, and angiogenesis. The same miRNA can affect multiple protein-coding genes, and the same gene can be affected by multiple miRNAs. The role of miRNA in tumorigenesis and development and research on its role in tumor diagnosis and as a prognostic marker and therapeutic target is expanding. miR-224 is not only abnormally expressed in a variety of tumors but also participates in the proliferation, differentiation, apoptosis, infiltration, and metastasis of tumor cells. It plays an important role in the occurrence and development of tumors. Moreover, it is related to the clinical characteristics and prognosis of tumors. It can affect the sensitivity of tumor cells to chemotherapy drugs. Murakami et al. [28] found that miR-224 is significantly expressed in liver cancer tissues compared to adjacent tissues. Wang et al. [29] found that the expression of miR-224 is negatively correlated with the expression of apoptosis inhibitor 5 (API5) in patients with hepatocellular carcinoma and finally confirmed that API5 is the target gene of miR-224. They further demonstrated that miR-224 can inhibit the expression of API5. Given that the latter is an antiapoptotic gene, miR-224 has been inferred to regulate API5 at the posttranscriptional level, thereby exerting an antiapoptotic effect. Consistent with Ni et al. [30], Song et al. [31] found that the expression of miR-224 in the lymph node tissues and cell lines of diffuse large B-cell tumors is lower than that in normal lymph node tissues and cell lines, and miR-224 is expressed at lower levels. The 5-year recurrence-free rate and overall survival rate of patients with low miR-224 expression were significantly lower than those of patients with high miR-224 expression. Therefore, miR-224 plays an important role in the occurrence and development of DLBCL and is related to patient prognosis.

PBX3 is closely related to tumorigenesis and development. PBX3 expression is positively correlated with the malignancy degree of prostate cancer [32]. PBX3 can reverse the inhibitory effect of Let-7c on colon cancer growth [33]. The high expression of PBX3 in colorectal cancer can be activated by mitogen-activated protein kinase/extracellular signal-regulated kinase signaling, which induces cell invasion and migration [34]. miR-33a-3p can reduce the invasion and migration capability of liver cancer cells through the targeted inhibition of PBX3. In this study, we found that PBX3, a target gene of miR-224, might mediate the promoting effect of the circNBPF10/miR-224 axis. circNBPF10, a ceRNA, limits the functional availability of miR-224 through sequence complementation. Therefore, our study revealed new aspects of the cellular and pathophysiological roles of circNBPF10 and miR-224, both of which could be considered as potential molecular targets for the treatment of lung cancer.

## 5. Conclusion

circNBPF10 was highly expressed in lung cancer. The high expression of circNBPF10 was related to distant metastasis and poor prognosis in patients with lung cancer. circNBPF10 targeted miR-224 to upregulate the expression of PBX3 and thus aggravated the malignant progression of lung cancer.

## Data Availability

The data used to support the findings of this study are available from the corresponding author upon request.

## Supplementary Materials

Supplementary Materials Figure S1. Relative expression of circNBPF10. Figure S2. The effect of circNBPF10 on the clone formation of lung cancer cells.
