# Supplementary figures and images for: circNBPF10/miR-224 Axis Regulates PBX3 to Promote the Malignant Progression of Lung Cancer
Source: J Oncol. 2022 Mar 17;2022:2832920. doi: 10.1155/2022/2832920 (PMC8947861; doi:10.1155/2022/2832920)

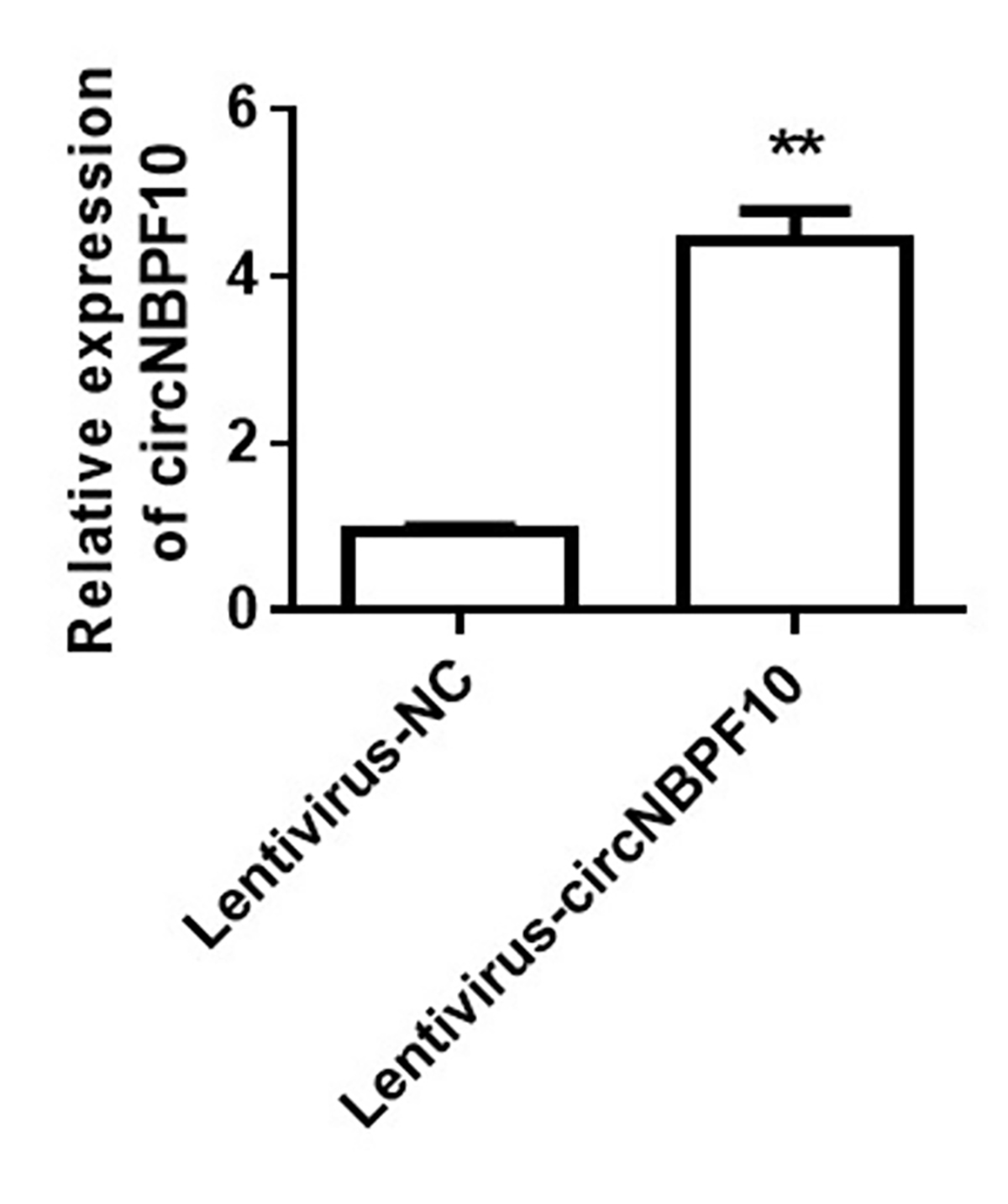

Supplement: Supplementary Materials — Figure S1. Relative expression of circNBPF10. Figure S2. The effect of circNBPF10 on the clone formation of lung cancer cells. [file 2832920.f1.zip › 2832920.f1/FigureS1.jpg]

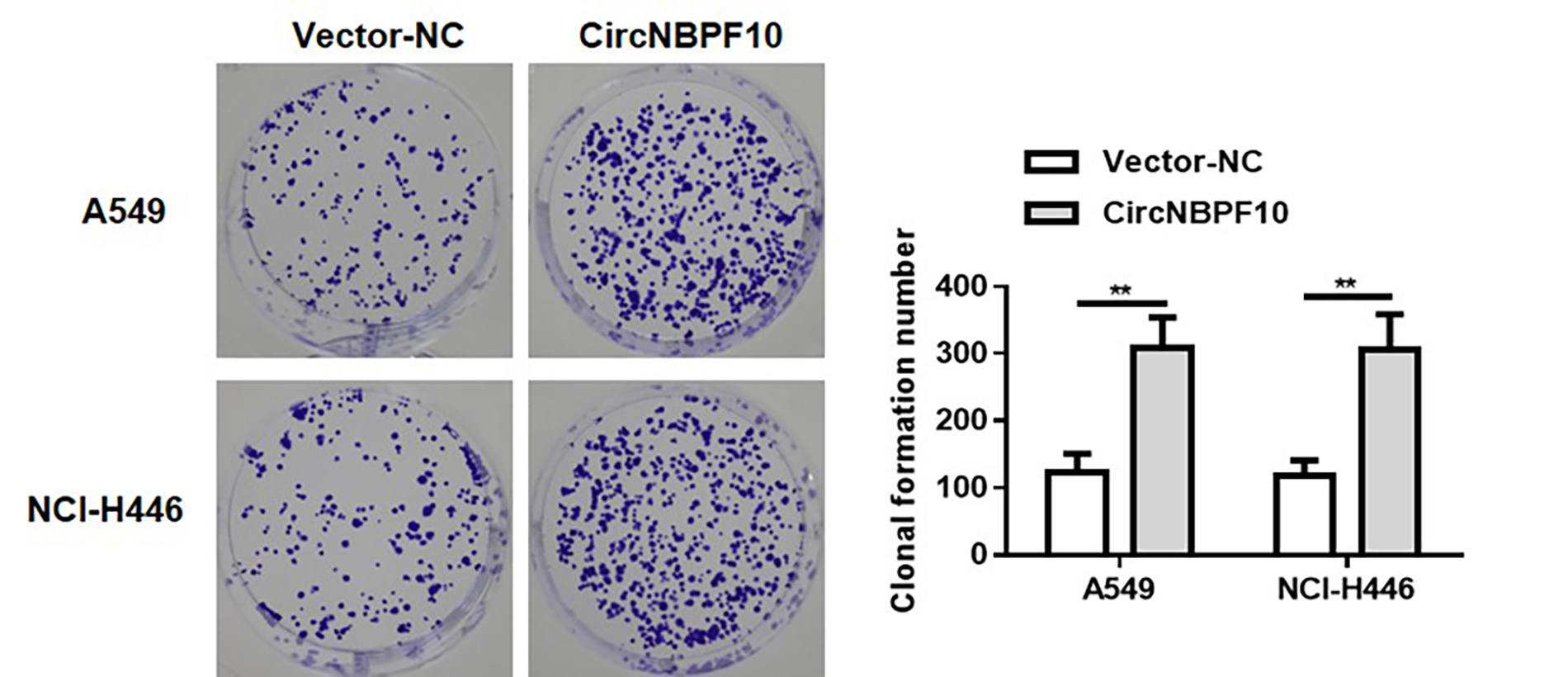

Supplement: Supplementary Materials — Figure S1. Relative expression of circNBPF10. Figure S2. The effect of circNBPF10 on the clone formation of lung cancer cells. [file 2832920.f1.zip › 2832920.f1/FigureS2.jpg]
